# Estratificação de Risco na Insuficiência Cardíaca: O Papel do Escore MAGGIC em uma População Brasileira

**DOI:** 10.36660/abc.20260305

**Published:** 2026-05-11

**Authors:** Evandro José Cesarino, Marildes Luiza de Castro, Larissa Neto Espíndola, Regina Celia Garcia de Andrade, Carolina Baraldi Araujo Restini

**Affiliations:** 1 Universidade de São Paulo Faculdade de Ciências Farmacêuticas de Ribeirão Preto Ribeirão Preto SP Brasil Universidade de São Paulo Faculdade de Ciências Farmacêuticas de Ribeirão Preto, Ribeirão Preto, SP - Brasil; 2 Associação Ribeirãopretana de Ensino, Pesquisa e Assistência ao Hipertenso Ribeirão Preto SP Brasil Associação Ribeirãopretana de Ensino, Pesquisa e Assistência ao Hipertenso (AREPAH), Ribeirão Preto, SP - Brasil; 3 Faculdade de Medicina da Universidade Federal de Minas Gerais Hospital das Clínicas Belo Horizonte MG Brasil Hospital das Clínicas, Faculdade de Medicina da Universidade Federal de Minas Gerais (UFMG), Belo Horizonte, MG - Brasil; 4 Hospital Santa Izabel Salvador BA Brasil Hospital Santa Izabel, Salvador, BA - Brasil; 5 Hospital Municipal de Salvador Salvador BA Brasil Hospital Municipal de Salvador, Salvador, BA - Brasil; 6 Michigan State University College of Osteopathic Medicine Michigan EUA Michigan State University College of Osteopathic Medicine, Michigan – EUA

**Keywords:** Insuficiência Cardíaca, Estudos de Validação como Tema, Avaliação de Risco, Estudo de Validação, Validade Social

A insuficiência cardíaca (IC) representa um dos principais desafios da cardiologia contemporânea e é a principal causa de internação por doença cardiovascular no Brasil, especialmente entre idosos.^1^ Além da alta carga de morbidade e mortalidade, a IC está associada a altas taxas de reinternação hospitalar e a um impacto significativo nos sistemas de saúde.^2^ Diante desse cenário, a identificação precoce de pacientes com maior risco de eventos adversos se tornou um componente fundamental do manejo clínico da doença.

As ferramentas de estratificação de risco desempenham um papel central nesse processo, auxiliando na estimativa do prognóstico, no planejamento terapêutico e na tomada de decisões clínicas compartilhadas com pacientes e familiares.

Diversos modelos prognósticos foram desenvolvidos para esse fim, incluindo o Modelo de Insuficiência Cardíaca de Seattle e o PREDICT-HF.^3,4^ No entanto, entre os escores disponíveis, o MAGGIC (*Meta-Analysis Global Group in Chronic Heart Failure*) se destaca por sua ampla validação e aplicabilidade clínica.^5,6^

Modelos desenvolvidos em populações específicas podem apresentar desempenho diferente em outros contextos clínicos, reforçando a importância da validação externa em diferentes contextos populacionais.^7^ No Brasil, apesar da alta prevalência de IC, estudos que avaliam o desempenho de modelos prognósticos amplamente utilizados na prática internacional ainda são escassos.

Nesse contexto, o estudo "Desempenho do Escore MAGGIC em Indivíduos com Insuficiência Cardíaca: Validação em uma População Brasileira" contribui de forma relevante ao avaliar o desempenho do escore MAGGIC em uma coorte brasileira de pacientes acompanhados em um ambulatório especializado em IC.^8^

No estudo, o escore MAGGIC apresentou boa capacidade discriminativa (AUC 0,72) e calibração adequada, resultados comparáveis aos observados em validações internacionais. Um aspecto particularmente relevante do estudo foi a análise estratificada por sexo, que demonstrou desempenho semelhante do modelo em homens e mulheres.

A consideração das diferenças específicas de sexo na IC tem recebido crescente atenção na literatura. Homens e mulheres apresentam diferenças importantes na epidemiologia, etiologia, remodelamento cardíaco e resposta ao tratamento.^9^ As mulheres, por exemplo, têm maior prevalência de IC com fração de ejeção preservada, enquanto os homens apresentam maior frequência de etiologia isquêmica e disfunção sistólica.^10^ Essas diferenças podem influenciar o desempenho dos modelos prognósticos, tornando a avaliação de sua precisão em ambos os sexos particularmente relevante.^11^

**Figura 1:**
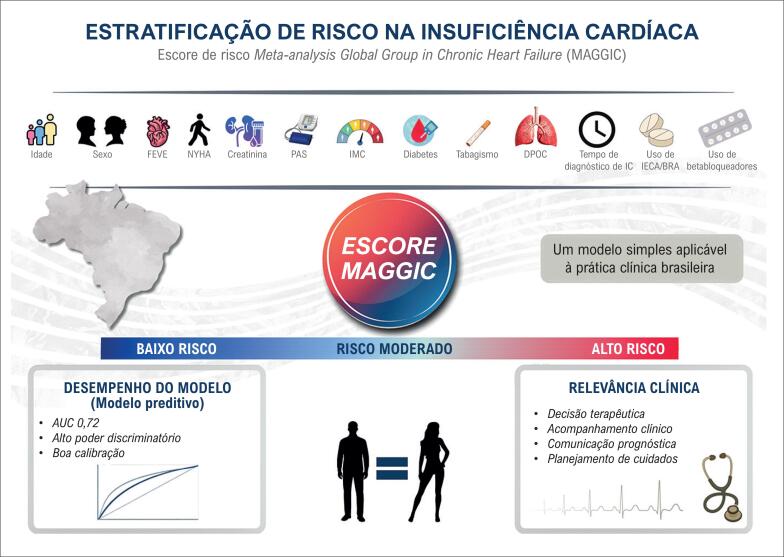
Validação da estratificação de risco de insuficiência cardíaca em brasileiros utilizando o escore MAGGIC.

Portanto, os achados do presente estudo reforçam evidências anteriores de que o escore MAGGIC apresenta desempenho consistente entre os sexos. Esse resultado tem implicações clínicas relevantes, sugerindo que o modelo pode ser aplicado com segurança à estratificação prognóstica de homens e mulheres com IC na população brasileira.^12^ Outro achado interessante foi a observação de discrepâncias entre a mortalidade prevista e a observada em algumas categorias de risco. Houve uma tendência à subestimação da mortalidade em grupos de menor risco e uma leve superestimação em pacientes de alto risco. Esse fenômeno já foi descrito em outras validações do escore e pode refletir diferenças nas características clínicas das populações avaliadas ou mudanças no manejo terapêutico ao longo do tempo^8^ (Figura 1).

Na prática clínica, o escore MAGGIC oferece uma vantagem significativa por utilizar apenas variáveis clínicas e laboratoriais amplamente disponíveis, tornando-o adequado para aplicação em diferentes contextos de saúde, incluindo ambientes com recursos limitados, uma realidade ainda presente em diversas regiões do Brasil.

Apesar das contribuições do estudo, algumas limitações devem ser consideradas. A análise foi conduzida em um único centro terciário, o que pode limitar a generalização dos resultados. Além disso, tanto a coorte analisada quanto o desenvolvimento original do escore ocorreram antes da ampla incorporação de terapias contemporâneas modificadoras do prognóstico, como os inibidores do cotransportador de sódio-glicose tipo 2 e o sacubitril valsartana, que demonstraram reduzir a mortalidade e as hospitalizações em pacientes com IC.^13^ A incorporação dessas terapias pode alterar o perfil de risco das populações atuais, reforçando a necessidade de reavaliar periodicamente o desempenho dos modelos prognósticos.

Em resumo, a validação do escore MAGGIC em uma população brasileira reforça sua utilidade como ferramenta de estratificação de risco na prática clínica. O desempenho consistente do modelo e a ausência de diferenças relevantes entre homens e mulheres corroboram sua aplicabilidade na população nacional. À medida que novas terapias modificadoras do prognóstico continuam a transformar o tratamento da IC, a integração de modelos prognósticos simples e validados, adaptados a diferentes realidades populacionais, permanecerá essencial para apoiar as decisões clínicas e otimizar o cuidado ao paciente.
