# Supplementary material for: Estratificação de Risco na Insuficiência Cardíaca: O Papel do Escore MAGGIC em uma População Brasileira
Source: Arq Bras Cardiol. 2026 May 11;123(3):e20260305. [Article in Portuguese] doi: 10.36660/abc.20260305 (PMC13398839; doi:10.36660/abc.20260305)
Supplement: Translation

# Translation: Risk Stratification in Heart Failure: The Role of the MAGGIC Score in a Brazilian Population

**DOI:** 10.36660/abc.20260305i

**Authors:** Evandro José Cesarino, Marildes Luiza de Castro, Larissa Neto Espíndola, Regina Celia Garcia de Andrade, Carolina Baraldi Araujo Restini

**Affiliations:** 1 Ribeirão Preto SP Brazil Universidade de São Paulo Faculdade de Ciências Farmacêuticas de Ribeirão Preto, Ribeirão Preto, SP – Brazil; 2 Ribeirão Preto SP Brazil Associação Ribeirãopretana de Ensino, Pesquisa e Assistência ao Hipertenso (AREPAH), Ribeirão Preto, SP – Brazil; 3 Belo Horizonte MG Brazil Hospital das Clínicas, Faculdade de Medicina da Universidade Federal de Minas Gerais (UFMG), Belo Horizonte, MG – Brazil; 4 Salvador BA Brazil Hospital Santa Izabel, Salvador, BA – Brazil; 5 Salvador BA Brazil Hospital Municipal de Salvador, Salvador, BA – Brazil; 6 Michigan USA Michigan State University College of Osteopathic Medicine, Michigan – USA

**Keywords:** Heart Failure, Validation Studies as Topic, Risk Assessment, Validation Study, Social Validity

Heart failure (HF) represents one of the main challenges in contemporary cardiology and is the leading cause of hospitalization for cardiovascular disease in Brazil, especially among older adults.^1^ In addition to the high burden of morbidity and mortality, HF is associated with high rates of hospital readmission and a significant impact on healthcare systems.^2^ Given this scenario, early identification of patients at higher risk of adverse events has become a fundamental component of the clinical management of the disease.

Risk stratification tools play a central role in this process, assisting in prognostic estimation, therapeutic planning, and shared clinical decision-making with patients and families.

Several prognostic models have been developed for this purpose, including the Seattle Heart Failure Model and PREDICT-HF.^3,4^ However, among the available scores, MAGGIC (Meta-Analysis Global Group in Chronic Heart Failure) stands out for its broad validation and clinical applicability.^5,6^

Models developed in specific populations may exhibit different performance in other clinical settings, reinforcing the importance of external validation in different population contexts.^7^ In Brazil, despite the high burden of HF, studies evaluating the performance of prognostic models widely used in international practice are still scarce.

In this context, the study "Performance of the MAGGIC Score in Individuals with Heart Failure: Validation in a Brazilian Population," makes a relevant contribution by evaluating the performance of the MAGGIC score in a Brazilian cohort of patients followed in a specialized outpatient clinic for HF.^8^

In the study, the MAGGIC score showed good discriminative ability (AUC 0.72) and adequate calibration, results comparable to those observed in international validations. A particularly relevant aspect of the study was the stratified analysis by sex, which demonstrated similar model performance in men and women.

Consideration of sex-specific differences in HF has received increasing attention in the literature. Men and women exhibit important differences in epidemiology, etiology, cardiac remodeling, and response to treatment.^9^ Women, for example, have a higher prevalence of HF with preserved ejection fraction, while men have a higher frequency of ischemic etiology and systolic dysfunction.^10^ These differences can influence the performance of prognostic models, making the evaluation of their accuracy in both sexes particularly relevant.^11^

Therefore, the findings of the present study reinforce prior evidence that the MAGGIC score performs consistently across sex. This result has relevant clinical implications, suggesting that the model can be safely applied to the prognostic stratification of men and women with HF in the Brazilian population.^12^ Another interesting finding was the observation of discrepancies between predicted and observed mortality in some risk categories. There was a tendency towards underestimation of mortality in lower-risk groups and slight overestimation in high-risk patients. This phenomenon has already been described in other validations of the score and may reflect differences in the clinical characteristics of the populations evaluated or changes in therapeutic management over time^8^ (Figure 1).

**Figure 1:** Validation of heart failure risk stratification in Brazilians using the MAGGIC score.

In clinical practice, the MAGGIC score offers a significant advantage because it uses only widely available clinical and laboratory variables, making it suitable for application in different healthcare settings, including resource-limited environments, a reality still present in several regions of Brazil.

Despite the study's contributions, some limitations should be considered. The analysis was conducted at a single tertiary center, which may limit the generalizability of the results. Furthermore, both the cohort analyzed and the score's original development occurred before the widespread incorporation of contemporary prognosis-modifying therapies, such as sodium-glucose cotransporter type 2 inhibitors and sacubitril valsartan, which have been shown to reduce mortality and hospitalizations in patients with HF.^13^ The incorporation of these therapies may alter the risk profile of contemporary populations, reinforcing the need to reassess the performance of prognostic models periodically.

In summary, the validation of the MAGGIC score in a Brazilian population reinforces its usefulness as a risk stratification tool in clinical practice. The model's consistent performance and the absence of relevant differences between men and women support its applicability in the national population. As new prognosis-modifying therapies continue to transform the treatment of HF, the integration of simple, validated prognostic models tailored to different population realities will remain essential for supporting clinical decisions and optimizing patient care.
